# The Association Between Vitamin D and Urinary Tract Infection in Children: A Case-Control Study

**DOI:** 10.7759/cureus.25291

**Published:** 2022-05-24

**Authors:** Sathya Chidambaram, Umapathy Pasupathy, Sangeetha Geminiganesan, Divya R

**Affiliations:** 1 Department of Paediatrics, Sri Ramachandra Institute of Higher Education and Research, Chennai, IND; 2 Paediatric Nephrology, Sri Ramachandra Institute of Higher Education and Research, Chennai, IND; 3 Paediatrics, Sri Ramachandra Institute of Higher Education and Research, Chennai, IND

**Keywords:** preschool children, cathelicidin, urinary tract infection, children, vitamin d

## Abstract

Background and objective

Urinary tract infection (UTI) is one of the common causes of febrile illness in young children. Vitamin D influences the levels of endogenous cathelicidin, an antimicrobial peptide, which improves bladder wall immunity and prevents UTIs. In light of this, we conducted this study to determine the association between vitamin D deficiency and UTIs in children and to identify whether vitamin D deficiency is one of the risk factors for UTIs.

Materials and methods

This was a case-control study of children aged between one to five years. Eighty-two children with the first episode of febrile culture-proven UTI as cases and 82 healthy children as a control group were included in this study. The sera were analyzed for 25-hydroxy vitamin D levels and classified as vitamin-D deficient if their level was below 30 ng/mL. Descriptive statistics were presented as numbers and percentages. Continuous data were expressed as means and standard deviations (SD). Pearson's chi-square test was used to test the significance of the differences in variables between the two groups. Multiple logistic regression equation methods were used to predict the relationship between the dependent and independent variables.

Results

The mean age of the study and the control group was 2.36 ±1.42 years and 2.57 ±1.26 years, respectively. The mean serum 25-hydroxy vitamin D levels in the patients and controls were 24.27 ±9.70 ng/mL and 31.97 ±10.7 ng/mL (p<0.001), respectively. Vitamin D deficiency was present in 34 (41.5%) patients and 10 (2.2%) in the control group (p<0.001).

Conclusion

Based on our findings, vitamin D deficiency might be one of the risk factors for UTIs in children. Vitamin D deficiency is significantly associated with febrile UTIs in children between one to five years of age.

## Introduction

Micronutrient deficiency in children is an evolving healthcare challenge, so far exhibiting only the tip of the iceberg in most developing countries including India [1]. Urinary tract infection (UTI) is one of the common causes of febrile illness in young children. Various known risk factors are involved in the pathogenesis of UTIs. If it is not diagnosed and treated promptly, it may lead to complicated UTIs and subsequently to renal scarring, proteinuria, and hypertension later in life [2]. Vitamin D - “the sunshine vitamin” - plays a critical role in calcium and phosphorous homeostasis and bone health. Moreover, it is also believed that vitamin D has a profound role in the overall health of several other organs as vitamin D receptors are found in them. The deficiency of vitamin D increases the risk of respiratory infections, hypocalcemic seizures, and growth disturbances, especially in preterm babies [3]. Vitamin D influences the levels of endogenous antimicrobial peptide, cathelicidin, thereby promoting bladder wall immunity and preventing UTIs. When the urinary tract is infected, our body produces endogenous antimicrobial peptides at the epithelial surfaces as the first line of defense. Cathelicidins and defensins are the two important antimicrobial peptides in humans as they cause membrane lysis by interacting with the microbial cytoplasmic membrane, inhibit biofilm formation, and modulate various immune responses. Vitamin D is an inducer of both human cathelicidin and β defensin-2. Hence vitamin D supplementation might help prevent UTIs in children and may also help in preventing their recurrence [4]. Based on this hypothesis, we conducted a study to determine the association between vitamin D and UTIs.

This article was previously presented as a meeting abstract at the Asian Congress of Paediatric Nephrology on March 31, 2021.

## Materials and methods

This was a case-control study conducted at a tertiary care center in South India from October 2018 to February 2021 covering all seasons. We obtained approval from the Institutional Research Ethics Committee, Sri Ramachandra University (REF: CSP-MED/18/AUG/45/120). The sample size was calculated based on the study by Mahyar et al. [2]; with an alpha error of 5% and power of 80%, 82 children aged between one to five years with the first episode of febrile culture-positive UTI who presented consecutively to our hospital were taken as cases. Group matching based on age and sex of the patients was applied to select 82 healthy children who presented to our hospital for elective surgeries without any urinary symptoms and were enrolled as the control group. Simultaneous sampling was done in both case and control groups till the desired sample size was achieved. Informed written consent was obtained from the parents of all participants in their own language before enrolment. UTI was defined as the growth of a significant number of organisms of a single species in urine. More than 100,000 colony-forming units (CFU)/mL in a mid-stream urine collection and more than 50,000 CFU/mL in a urethral catheterization sample were considered as significant growth of organisms [5].

Children with vesicoureteral reflux, anatomical abnormality of the kidneys, ureter, and bladder, children with metabolic bone disease, those with any clinical features of rickets, those diagnosed with chronic systemic diseases, those on vitamin D supplementation, and children with malnutrition were excluded from the study. Clinical history indicative of UTIs, such as fever, vomiting, abdominal pain, dysuria, urgency, and frequency, were documented along with examination findings. Baseline investigations included urinalysis, urine culture, complete blood count, and abdominal ultrasound. Two milliliters of peripheral venous blood were drawn from the patients and controls. The sera were analyzed for 25-hydroxy vitamin D levels using UniCel DxI chemiluminescent immunoassay system (Beckman Coulter Inc., Brea, CA) and values were expressed as ng/mL. 25-hydroxy vitamin D value less than 20 ng/mL was considered vitamin D-deficient, 20-30 ng/mL as insufficient, and more than 30 ng/mL as normal vitamin D levels [6]. Children who were found to have vitamin D deficiency were treated with 60,000 IU of vitamin D weekly for six weeks and 600 mg of calcium daily as per Indian Academy of Pediatrics (IAP) guidelines [7].

Statistical analysis was performed using SPSS Statistics, version 17 (IBM, Armonk, NY) for Microsoft Windows. Descriptive statistics were presented as numbers and percentages. Continuous data were expressed as means and standard deviations (SD). Pearson's chi-square test was used to test the significance of the difference between the variables in the two groups. Multiple logistic regression equation methods were used to predict the relationship between the dependent and independent variables. Unadjusted and adjusted odds ratios from logistic regression were used as a measure of effect. The analysis of variance (ANOVA) test was used to compare continuous variables. A two-sided p-value <0.05 was considered to indicate statistical significance.

## Results

Among the patients, 62 children (75.6%) were aged between one to three years and 20 (24.4%) were between three to five years. In the control group, 60 (73.2%) were aged between one to five years and 22 (26.8%) were between three to five years. The mean age of the case and control group was 2.36 ±1.42 years and 2.57 ±1.26 years, respectively. In the case group, 37 (45.1%) were male and 45 (54.9%) were female. In the control group, 49 (59.8%) were male and 33 (40.2%) were female (Table 1). All patients had a fever. Other common symptoms were vomiting (n=30; 36.5%), crying during micturition (n=34; 41%), and pollakiuria (n=23; 28%). Escherichia coli (E. coli) was the most common organism isolated (n=77; 93%) followed by Proteus (n=3; 3.6%), Morganella (n=1; 1.2%), and Acinetobacter (n=1; 1.2%). Abdominal ultrasound was done for all children with UTI, of which 21 (54.8%) had abnormal findings such as bladder wall thickening, cystitis, and increased internal echoes.

**Table 1:** Characteristics of patients and controls

Variables		Cases, n (%)	Controls, n (%)	P-value
Age	1–3 years	62 (75.6%)	60 (73.2%)	0.23
	3–5 years	20 (24.4%)	22 (26.8%)	
Sex	Male	37 (45.1%)	49 (59.8%)	0.39
	Female	45 (54.9%)	33 (40.2%)	
Gestational age at birth	Term	67 (61.7%)	60 (73.2%)	0.19
	Preterm	15 (18.3%)	22 (26.8%)	
Breastfeeding history	Breastfed	29 (35.4%)	28 (34.1%)	0.8
	Not breastfed	53 (64.6%)	54 (65.9%)	

**Figure 1:**
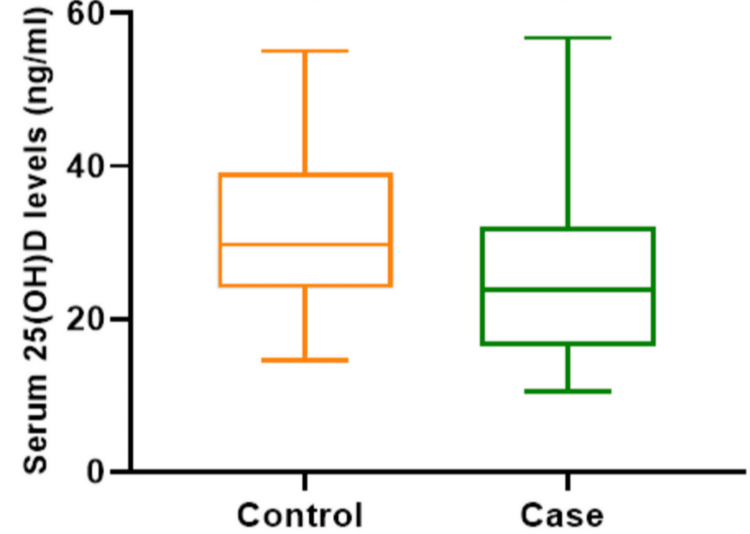
Box and whisker plot of vitamin D levels among cases and controls

Normal vitamin D levels were documented in 22 (26.8%) patients and 40 (48.8%) controls. Among the patients, 34 (41.5%) had vitamin D deficiency and 26 (31.7%) had vitamin D insufficiency. In the control group, 10 (12.2%) had vitamin D deficiency and 32 (39%) had vitamin D insufficiency (Table 2). The mean serum 25-hydroxyvitamin D levels in the patients and the controls were 24.27 ±9.70 ng/mL and 31.97±10.7 ng/mL, respectively. There was a statistically significant difference in vitamin D levels between the two groups (p<0.001). Applying the box and whisker plot also showed that the mean, upper, and lower vitamin D levels among the patients were significantly lower compared to those in the controls (Figure 1). There was no significant correlation between vitamin D levels and age, hemoglobin, total WBC count, and neutrophilia. Multivariate logistic regression analysis was done to assess the risk factor for UTIs in the study population, such as vitamin D level, female gender, preterm babies, and children who were not breastfed exclusively for six months. Children with vitamin D levels less than 20 ng/mL were at 2.48 times higher risk of getting UTI than children who had normal vitamin D levels (adjusted odds ratio: 2.486; 95% confidence interval: 1.610-3.838; p<0.001) (Table 3).

**Table 2:** Comparison of vitamin D levels between patients and controls

Vitamin D levels	Cases, n (%)	Controls, n (%)	Total, n (%)	P-value
Deficiency	34 (41.5%)	10 (12.2%)	44 (26.8%)	<0.001
Insufficiency	26 (31.7%)	32 (39%)	58 (35.4%)	
Normal	22 (26.8%)	40 (48.8%)	62 (37.8%)	
Total	82 (100%)	82 (100%)	164 (100%)	

**Table 3:** Multivariate logistic regression analysis UTI: urinary tract infection

Risk factors for UTI	Adjusted odds ratio	95% confidence interval	P-value
Vitamin D level less than 20 ng/mL	2.486	1.610–3.838	<0.001
Female child	0.42	0.27–1.10	0.06
Preterm baby	1.34	0.55–2.99	0.19
Not breastfed exclusively	0.77	0.41–1.56	0.91

## Discussion

The occurrence of UTI in children follows a bimodal distribution with the initial peak in the first year of life and another peak at the age of toilet training between two and three years. The risk of having a UTI before the age of 14 years is 1-3% in boys and 3-10% in girls. The overall prevalence of UTIs among febrile infants and young children is estimated to be 4-7%. However, it varies according to age, sex, race, nutritional status, state of circumcision, and other factors [7-10]. UTI is more common in female children, due to shorter female urethra, and male infants with phimosis. During toddler years, toilet training can lead to volitional holding and bladder stasis, promoting UTIs [11-12]. The mean age of the patients and control group in this study was 2.36 ±1.42 and 2.57 ±1.26 years, respectively. In the case group, 37 (45.1%) were male and 45 (54.9%) were female. In the control group, 49 (59.8%) were male and 33 (40.2%) were female. A similar age group was studied by Shalaby et al. in which females constituted 60% of the group [13].

Most pediatric UTIs are caused by Gram-negative coliform bacteria that arise from fecal flora colonizing the perineum, and entering and ascending the urinary tract.E. coli* *is the most common uropathogen, responsible for approximately 80% of pediatric UTIs. Other common uropathogens include Klebsiella, Proteus, Enterobacter, and Enterococcus species [14-15]. Similarly, in our study, 93% of the children had E. coli growth in urine culture. Vitamin D has a role in the prevention of UTIs in children. The association between vitamin D levels and many infectious diseases has been well known for a long time [16]. Studies have shown that uropathogenic E. coli strains that are resistant to human cathelicidin are more prone to invade the upper urinary tract than susceptible strains. Sufficient concentrations of circulating vitamin D are mandatory for optimal cathelicidin production by macrophages [17].

Hertting et al. reported that vitamin D3 supplementation enhances the production of cathelicidin in UTIs [18]. Our study also explored the association of febrile UTIs in children with vitamin D levels. Yang et al. observed that serum 25-hydroxyvitamin D level <20 ng/mL was associated with a significantly increased incidence of UTI in infants (p<0.012). He also concluded that vitamin D supplementation had decreased the risk of developing UTI in his study population (p=0.001) [19]. Shalaby et al. reported that vitamin D deficiency is an independent risk factor for UTIs in children [13]. Tekin et al. also reported that vitamin D level <20 ng/mL was significantly associated with an increased risk of UTI (p<0.001) [20]. In our study group, the mean serum 25-hydroxy vitamin D level among the 132 cases was 24.27 ±9.70 ng/mL and the difference between the control group was statistically significant (p<0.001). Also, children with vitamin D levels <20 ng/mL had a 2.48 times higher risk of getting UTI. However, we did not study the risk of UTI after the supplementation of vitamin D. Georgieva et al. observed in their study that serum vitamin D levels negatively correlated with age and were significantly lower in girls [21]. However, in our study, there was no statistically significant difference in vitamin levels in terms of age, gender, and breastfeeding.

In contrast to all the above studies, Karatekin et al. reported that vitamin D supplementation had increased the risk of UTI in formula-fed infants by up to 76% [22]. Övünç Hacıhamdioğlu et al. found that patients with a UTI and sufficient levels of vitamin D had significantly higher urine cathelicidin levels than controls with insufficient vitamin D levels (p=0.001) [23].

The major limitation of the current study is that we did not evaluate cathelicidin levels in our study population. Hence, further studies with larger cohorts are essential in the Indian subcontinent among people from varied geographic regions, and with varying ethnic, dietary, and cultural practices.

## Conclusions

Children with vitamin D deficiency are at risk of getting UTIs. Vitamin D might have a major role in the pathogenesis of the first episode of UTI. Adequate supplementation of vitamin D may prevent UTIs in children.
